# Persons Experiencing Housing Instability Perspectives on Medicaid Managed Care Organizations and Homeless Shelters

**DOI:** 10.1177/24731242251371428

**Published:** 2025-08-27

**Authors:** Emily R. Clear, Allison M. Scott, Kelsie Kwok, Mark A. Ribott, Teresa M. Waters, Rachel Hogg-Graham

**Affiliations:** ^1^ Department of Health Management and Policy, College of Public Health, University of Kentucky, Lexington, Kentucky, USA.; ^2^ Department of Communication, University of Kentucky, Lexington, Kentucky, USA.; ^3^ Institute for Public and Preventive Health, Augusta University, Augusta, Georgia, USA.

**Keywords:** persons experiencing housing instability, Medicaid managed care organizations, homeless

## Abstract

**Background:**

Persons experiencing housing instability (PEHIs) are medically vulnerable and at increased risk for poor health outcomes, high clinical service utilization, and mortality. Unstable housing is just one of many social determinants of health or nonmedical factors influencing health outcomes.

**Methods:**

Focus groups were conducted on-site at two Kentucky homeless shelters to assess the structure and perceived effectiveness of Medicaid managed care organizations (MCOs) and community-based organizations (CBOs) partnerships. We share perspectives of homeless Medicaid enrollees who are living without housing on the interaction between Medicaid MCOs and homeless shelters addressing unmet social needs.

**Results:**

Three themes emerged from our qualitative analysis: (1) Benefits of and barriers to receiving various services through Medicaid, (2) Medicaid does not appear to interface well with community-based shelters, and (3) Medicaid enrollees living without housing perceive a lack of information from Medicaid. Concerns raised by participants included barriers to receiving services, strengthening resource and referral processes, and increasing communication with both CBOs and Medicaid enrollees. These concerns must be addressed to improve care and outcomes.

**Conclusions:**

PEHIs rely on homeless shelters to help them enroll and utilize Medicaid rather than relying on Medicaid to identify and utilize CBOs. There are opportunities for improvement in how MCOs interact with PEHI enrollees. PEHIs utilize Medicaid and navigate cross-sector relationships in different ways than other Medicaid enrollees.

## Introduction

Persons experiencing housing instability (PEHIs) are medically vulnerable, and their lack of insurance can lead to greater utilization of the emergency department, which is often fragmented and expensive.^1^ On a single night in 2022, ∼582,500 people experienced sheltered homelessness.^2^ Of these individuals, 37% identified as minority race/ethnicity, and 50% of those experiencing sheltered homelessness with families/children identified as minority. Many PEHIs have been able to gain coverage through Medicaid but tend to have higher health care needs compared with Medicaid beneficiaries who are not experiencing homelessness.^3^ Housing instability is just one of many social determinants of health (SDOH) or nonmedical factors, influencing health outcomes.^4^ The unmet social need for housing can worsen existing health issues because of difficulty providing self-care or seeking medical care.^5^ PEHIs are at increased risk for poor health outcomes, including communicable diseases, cardiovascular disease,^6^ environmental exposures, and mental health disorders. Chronic homelessness has been linked to high clinical service utilization and increased mortality incidence.^7,8^

Medicaid managed care organizations (MCOs) are well-positioned to address the unmet social needs of PEHIs. PEHIs are able to apply for Medicaid in person, over the phone, or online. The application requires the applicant to submit their demographic information and a mailing address, which proves challenging for PEHI. With respect to housing instability, many MCOs screen beneficiaries for housing needs and hire or leverage housing coordinators to facilitate access to housing. U.S. Centers for Medicare & Medicaid Services has now clarified that Medicaid plans can offer community support “in lieu of services” to provide housing support and hire staff to assist beneficiaries in obtaining and sustaining housing, including transitional housing. Some states now require that MCOs work with housing authorities.^9,10^ As a result, many MCOs are partnering with community-based organizations (CBOs), including shelters, to address housing insecurity.^11^

An estimated 69% of all Medicaid beneficiaries were enrolled in Medicaid MCO plans in 2020.^12^ With two-thirds of Medicaid enrollees covered by a state-contracted MCO, these organizations have a uniquely powerful opportunity to screen for and assist with enrollee unmet social needs through referrals to CBOs. The International Classification of Diseases Version 10 coding (ICD-10 codes) offers MCOs and providers a systematic approach to capturing patient social needs.^13^ Specifically, ICD-10-CM Code Z59^14^ includes problems related to housing and economic circumstances, including homelessness and inadequate housing.^14^ Dapkins and Blecker reported that Medicaid recipients with an ICD-10-CM code for homelessness had higher rates of interrupted Medicaid coverage compared with those who did not have an ICD-10-CM homelessness diagnostic code, impacting both enrollees and the systems created to help them.^15^

While many PEHIs are eligible to enroll in Medicaid, the process for gaining and maintaining coverage has significant limitations. Barriers such as not having a mailing address, transportation, or access to technology, and a lack of information on what plan(s) would best fit their needs may hinder some from enrolling in Medicaid.^1^ Limited literacy, unstable contact information, and mental health conditions also contribute to problems in the enrollment process. PEHIs are “distrustful of public systems and reluctant to apply for assistance.”^16^ Helping individuals overcome this disengagement often requires significant time, effort, and relationship building.^16^

SDOHs are a critical driver of health outcomes, especially in Medicaid populations. MCOs can partner with homeless shelters to better meet the needs of their beneficiaries with insecure housing. However, little is known about these partnerships from the perspective of the PEHIs. To optimize the effectiveness of MCO–CBO partnerships, we must first learn how these partnerships impact the PEHIs that the partnerships serve. The purpose of the present study was to share the perspective of PEHI Medicaid enrollees on the interaction between Medicaid MCOs and homeless shelters. Understanding these perspectives will allow policymakers, MCOs, and CBOs the opportunity to design systems of care that are more responsive to beneficiary needs.

## Methods

Focus groups with Medicaid enrollees experiencing homelessness were conducted to assess the structure and perceived effectiveness of MCO–CBO partnerships, including experiences with the process of being referred to a CBO, satisfaction of needs met, barriers for accessing CBO services, and ways to improve the system of service referrals. Two homeless shelters in two separate urban areas in a Southeastern state participated. Researchers worked with shelter contacts to recruit participants, and shelter employees let homeless shelter guests know about the opportunity to participate in a focus group, posted recruitment materials, and communicated dates and times. Dinner was provided to all shelter guests, regardless of whether they participated or not, as a thank you for the access to the population. Researchers coordinated with both shelters on logistics and the need to feed all shelter guests on the evening when focus groups were conducted. Providing dinner to all shelter guests was intended to demonstrate that participation was not coercive and foster trust, with the hope they would speak with us openly and honestly.

Inclusion criteria included individuals currently or previously enrolled in Medicaid. Almost all homeless shelter guests are qualified. The focus group facilitator and qualitative expert read the consent form to all participants at the beginning of each focus group. Participants had the opportunity to ask questions before verbally consenting. Focus groups were not audio- or video-recorded to protect participant anonymity and maintain the trust of participants. Responses were not recorded, and there was a waiver of documentation of informed consent to honor vulnerability, make participants feel more comfortable, increase their willingness to provide honest answers reflecting both positive and negative experiences, and decrease the fears of social stigma and confidentiality. Only the focus group facilitator and the note-taker were present for interviews to keep the research footprint small. The note-taker was asked to take notes on each session, and, as much as possible, she wrote participant statements word for word to best capture focus group content. Guided by a discussion script, the focus group facilitator asked participants to share and evaluate their experiences with the homeless shelter and Medicaid MCOs partnerships. Questions for enrollees focused on (1) their experience with the referral process, (2) the appropriateness of the shelter for addressing needs, (3) satisfaction with prioritization of their needs by the shelter, (4) barriers and facilitators to accessing health care services, and (5) what could be improved with the system of service referrals to the shelter, tracking, and follow-through based on their experience.

Two focus groups were conducted at each of the two shelters, for a total of four focus groups. Each focus group’s size ranged from 7–9 participants, with a total sample size of 32. Demographic information was not collected, and the Institutional Review Board at the University of Kentucky approved this research, IRB protocol# 75572. We worked closely with community partners to access the homeless population, and our decision not to collect demographic information or audio record participant responses was based on input from the shelter staff’s recommendation on how best to collect data in this context and our study advisory board. Conducting on-site focus groups with homeless shelter guests removes transportation, and technology barriers are often associated with hosting focus groups in person or virtually.

### Data analysis

Members of the research team analyzed the transcribed participant quotations using qualitative descriptive analysis,^17^ which is an inductive and low-inference method of gaining an understanding of a phenomenon through the everyday terms of those who are stakeholders. This method was particularly appropriate given that our goal was to learn about the participants’ experience in their own words without layering interpretive lenses on top of the data. We also followed the ENTREQ checklist of analysis items for enhancing transparency in reporting qualitative research.^18^

Data were analyzed in two stages. First, three coders (A.M.S., E.R.C., and M.A.R.) independently coded the data line by line using open coding in which salient themes were identified.^19^ During this first round of coding, the three coders revised the transcripts and identified categories of experiences, labeling the categories with phrases from the participants’ reports. The three coders then met to discuss and reach a consensus on integrating the themes into a comprehensive categorization scheme that accounted for every coder’s categories. After this, the coders consulted with two other authors (R.H.-G. and T.M.W.) to create a comprehensive categorization scheme that included all the coders’ individual categories. That is, we organized the “free codes” into a set of descriptive themes.^18^

In the second round of coding, the same three coders returned to the data to independently conduct the additional focused coding.^19^ The three coders met again to compare findings and finalize themes and subthemes. Specifically, the three coders worked together to ensure that every statement in the transcripts was clearly accounted for by the coding scheme. This step in the process allowed us to conduct further interpretation to develop final analytical themes.^18^ After this, all five authors conferred and reached a consensus through discussion to select representative quotations to illustrate each theme. While we conducted additional coding for the overall study (which was part of a broader project studying Medicaid MCO–CBO partnerships), the results and quotations presented below are the outcome of the qualitative descriptive analysis process.

## Results

Several primary themes emerged from our analysis: (1) Benefits of and barriers to receiving various services through Medicaid, (2) the perception that Medicaid does not interface well with community-based shelters, and (3) the perception that Medicaid enrollees experiencing unstable housing lack information that they need from Medicaid.

### Theme 1. Benefits of and barriers to receiving services through Medicaid

Many focus group participants reported experiencing some benefits (health and otherwise) to receiving health care through their Medicaid MCO, including free prescriptions and limited out-of-pocket expenses. As one enrollee reported, “[Named Medicaid Managed Care Organization] took care of my heart attack. The benefits package always good. I had my bypass done, they pay for it.” Participants also highlighted several nonmedical benefits received through their MCOs, such as gift cards. While some nonmedical benefits seemed uniform across MCOs, for example, cell phones, it was clear there was also variability across insurers. One participant stated, “I recently learned [Named Medicaid Managed Care Organization] helps with utilities and $1k for college.” One participant noted that they periodically check in to see what they qualify for, “Every time I go to the doctor, I call to see if I can get a gift card.” Another participant from the same focus group expressed appreciation for these benefits by stating, “It makes a difference when you have a insurance company that is givin’ you that much rewards.”

Participants were also quick to list several barriers to accessing health care through Medicaid. One common barrier reported by participants was access to dental care. Enrollees shared their experiences with Medicaid dental coverage, “You ain’t got good dental.” One participant elaborated, “With teeth, they consider it an elective surgery. So they don’t cover it. You gotta go to prison to get it fixed.” Several participants expressed the concern that Medicaid did not actually give them health care coverage, explaining that “a lot of people don’t take Medicaid, what can we use it for?” Limitations to having Medicaid included medications (“Only thing they might get you is ibuprofen”), finding a physician (“I ain’t got no doctor”), and out-of-pocket expenses (“Still gotta put down a $20, should get some free meds, no $10, $20 with it. Should be all free”). Lack of Medicaid coverage was particularly salient for participants, so the barrier to accessing Medicaid and the lack of help in navigating access were frustrating for some participants. “I think Medicaid should cover anything anyone has a problem with to get them back up to be productive. Some of it barely covers us to get around. Medicaid should cover what it takes to get up and be productive.” Another participant stated, “Every American should be covered by some kind of basic medical assistance from womb to tomb.” One participant in this group shared that the homeless shelter “helped me get a job. Ain’t none of this through Medicaid.”

### Theme 2. Medicaid does not interface well with community-based shelters

Based on participant reports, Medicaid does not appear to interface well with homeless shelters. One salient example of this that surfaced in multiple focus groups is that participants learned about the homeless shelter from sources other than Medicaid. For example, one participant said, “[Named Medicaid Managed Care Organization] connected me,” but this was the only person who said that Medicaid was involved in their shelter referral. All other participants in the four focus groups reported that they became connected to the homeless shelter through referrals through channels other than Medicaid. Several participants noted that they learned about the shelter mostly from word of mouth (“From a person who knew about it,” “From the right club”). Several participants found out about the shelter from their place of worship: One participant learned about the shelter “From the pulpit,” another said, “My church told me about it,” and yet another said they heard about the shelter from “someone from my prayer group from [Named Medicaid Managed Care Organization].” One participant shared, “I came from the Department of Corrections. They led us right here. My PO said, ‘We will willingly send you.’” Similarly, another participant said, “Police told me, ‘You go to the shelter, ask people down there.’ Worked my way, found the shelter.” Participants in one of the focus groups recognized the absence of Medicaid’s referral to the shelter. One participant said, “No one at Medicaid is telling you about this shelter.” This prompted a conversation that “Medicaid should be here to help.” and “Medicaid don’t tell you nothing.”

The lack of interfacing between Medicaid and community-based shelters led some Medicaid enrollees to seek resources on their own, as one participant described, “I’m out here and asking who has the best benefits. It’s me asking what benefits they’re getting. I look it up online and see benefits online on my phone.” One participant said that he relied on the homeless shelter to help him with Medicaid, not the other way around: “At a place like this, [there should be] a poster directing people how to enroll in Medicaid, how to do it.” One participant summarized the relationship between Medicaid and homeless shelters: “Reality is they are two separate things and don’t know how they interact.”

### Theme 3. Medicaid enrollees lack information from Medicaid

We found that Medicaid enrollees often experience confusion due to a lack of information about Medicaid. Participants said, “You got to choose between carriers and don’t know which cover what.” Another participant shared, “No one helps you pick and hope for the best.” Participants described the difficulty of finding information about or from Medicaid or connecting with any representative who can help them. One participant shared, “[State] has [Named Medicaid Managed Care Organization], so going to get it, I can’t find no representative here. I call on the phone and they ask me the same question 40 times. If you call [Named Medicaid Managed Care Organization], you have to talk on the phone for 60 min. Two different people ask the same question over and over. It’s a mess; I can’t do it.” One specific way Medicaid enrollees experienced a lack of information from Medicaid was regarding their Medicaid cards. Participants in one focus group expressed frustration not being able to have a physical card with participants sharing, “It’s a lot of stuff to know. If I ain’t know the stuff they said, I ain’t got no card.” and another responding, “I want to know why I ain’t got no card.” Two participants expressed exceptions to the lack of information, with one saying, “I get text notifications,” and the other saying, “I constantly get messages in my email.” Table 1 includes additional quotes by theme.

**Table 1.** Themes

Theme	Quote
Benefits and barriers	
Benefits received	“[MCO] gives me $400 every quarter, $4K for hearing aids, $2K for dental, $500+ for flex card.”
	“I recently learned [Named Medicaid Managed Care Organization] helps with utilities and $1K for college.”
	“[Named Medicaid Managed Care Organization] bought me a cell phone yesterday.”
	“I was in rehab and they paid for it.”
	“Last December this knee swolled up so bad, went to hospital for 6 days. I got [Named Medicaid Managed Care Organization], prescriptions free, clinic, no copay.”
	“[Named Medicaid Managed Care Organization] took care of my heart attack. The benefits package always good. I had my bypass done, they pay for it.”
Barriers to dental access	“With teeth they consider it an elective surgery. So they don’t cover it. You gotta go to prison to get it fixed.”
	“You ain’t got good dental.”
Medicaid did not give health care coverage	“Gotta do all this shit to get a doctor.”
	“I ain’t got no doctor.”
	“Still gotta put down a $20, should get some free meds, no $10, $20 with it. Should be all free.”
Medicaid does not appear to interface well with homeless shelters	
How you were referred the shelter	“Word of mouth.”
	“My dad brought me.”
	“A big billboard.”
	“When I came to area, my friend tried to help me out, went shelter to shelter, they referred me here. I’m grateful.”
Absence of Medicaid’s referral to homeless shelter	
	“Medicaid don’t tell you nothing.”
	“Who told you about this?”
	“I’m out here and asking who has the best benefits. It’s me asking what benefits they’re getting. I look it up online and see benefits online, on my phone.”
Lack of information about Medicaid	
	“Difficult part of [Named Medicaid Managed Care Organization]? Lack of communications. Nothing.”
	“How do you reach out?”
	“4 hours a day on the phone.”
	“[Named Medicaid Managed Care Organization] helps with utilities. Until I got that letter I didn’t know bonus benefits. It would be helpful if someone said ‘This what you get.’”
	“Nice to be told ‘We working with your case worker, if you missin’ on utilities, [Named Medicaid Managed Care Organization] can help what that.’ I only got a letter.”
	“I didn’t even know all that until just now.”
Medicaid cards	
	“Show they the card! I ain’t got no card.”
	“You got one?”
	“I ain’t never got a card.”
	“I’ve never had a card.”
	“They don’t’ do that. No paper. No card.”
	“Not a whole lot of people got a card.”

## Discussion

The lived experiences of Medicaid enrollees experiencing homelessness were solicited, including their perspectives on how the homeless shelter and Medicaid interface. Our data highlight the potential impact of community-based partners in meeting the needs of PEHI. Major study themes revealed that participants experienced benefits and barriers in receiving Medicaid services, but because Medicaid MCOs do not interface well with homeless shelters, which entails a lack of information regarding Medicaid, participants experienced confusion about the services available to them and reported that they relied on homeless shelters to get them connected with Medicaid services, rather than Medicaid connecting them with housing services.

We found that CBOs, rather than MCOs, referred participants of this study to homeless shelters. Participant responses underscore the need to explore the relationships and referral process between MCOs and CBOs. Our findings support previous research that reported the majority of social need referrals were among CBO–CBO partnerships, suggesting strong ties between these organizations, rather than their clinical or payer partners.^20^ In other words, participants reported that they wish Medicaid had referred them to the shelter, but instead, they found out about the shelter through other channels. Results from this study support previously reported suggestions from CBOs for a specific contact to coordinate referrals, overall misalignment with referral capacity, and the importance of bringing CBOs into the conversation early on screening and referrals to increase community carrying capacity.^20^ There is a limit on the load CBOs can absorb referrals from clinical systems,^21^ which impacts the community’s carrying capacity. CBOs have close-knit networks,^22^ resulting in connectivity and strength, but in the case of the PEHIs we interviewed, this connectedness did not extend to Medicaid MCOs.

Insights gained from the perspective of PEHIs can help inform future policies impacting those with unmet social needs. Participant reports of housing conditions and their impact as an unmet social need suggest there is room for improving Medicaid referrals and providing beneficial information to Medicaid enrollees through CBOs. Social needs, including homelessness, increased during the COVID-19 pandemic.

### Limitations

While we provide insight into the needs of the PEHI population, our small sample size limits generalizations related to other homeless shelters or CBOs settings. Providing food to all shelter guests aimed to avoid influencing participation and ensured self-selection in the focus groups. Our findings may be unique to the participants in this study, and future studies could prospectively explore the experiences of a broader range of Medicaid enrollees living with homelessness to explore how they are referred to and utilize Medicaid coverage and CBOs to address their health and social needs.

One strength of this research was the ability to successfully engage with and solicit input from Medicaid enrollees experiencing homelessness, which can be attributed to a number of study design choices. Hosting in-person focus groups at the homeless shelters during dinner service maximized the number of individuals who considered participating. We also believe that anonymity contributed to our ability to solicit honest and open responses to gain a deeper understanding of participants’ needs and perceived barriers.

### Health equity implications

Ensuring equitable health outcomes often requires addressing social needs. Persons living with unmet social needs face significant barriers to obtaining care for existing health care conditions and often develop additional medical needs. In this study, we focused on individuals who face housing insecurity, among other social needs, and our work offers unique insights regarding challenges faced by this population and highlights the need for participant-informed approaches to service delivery. To improve care and outcomes, Medicaid MCOs should address the reported barriers to receiving services, strengthen resource and referral processes, and increase communication with both homeless shelters and enrollees. Additionally, our study highlights the importance of CBOs such as homeless shelters and their trusted presence in the community as a vehicle for service delivery and partnership.^23^ Participants repeatedly noted that homeless shelters are an important source of information and assistance regarding Medicaid and its activities.

## Conclusions

While PEHIs qualify for Medicaid, they are not always aware of the services offered, and many report barriers to accessing various services through Medicaid. There are opportunities for improvement in the ways Medicaid MCOs interface with their enrollees and homeless shelters. Homeless shelter guests reported that they rely on the shelters to enroll in and utilize Medicaid, rather than relying on Medicaid to identify and utilize community-based shelters. Future research should aim to bridge the research gap between PEHIs, MCOs, and strengthen the coordination of care for enrollees as they move through organizations.

## Abbreviations Used

CBOs: Community-based organizations
ENTREQ: Enhancing transparency in reporting the synthesis of qualitative research
ICD-10 codes: The International Classification of Diseases Version 10 coding
MCOs: Medicaid managed care organizations
PEHIs: Persons experiencing housing instability
